# Divergences between the Brazilian national information systems for recording deaths from venomous animals

**DOI:** 10.1590/1678-9199-JVATITD-1430-18

**Published:** 2019-05-20

**Authors:** Rosany Bochner, Claudio Mauricio Vieira de Souza

**Affiliations:** 1 Oswaldo Cruz Foundation - Fiocruz. Institute for Scientific and Technological Information and Communication - ICICT, Rio de Janeiro, RJ, Brazil.; 2 Vital Brazil Institute, Niterói, Rio de Janeiro, RJ, Brazil.

**Keywords:** information system, death, SINAN, SIM, snake, spider, scorpion, bee

## Abstract

**Background:**

This paper aims to highlight and analyze discrepancies in reporting of deaths due to venomous animals in Brazil, from 2001 to 2015, between two national information systems: The Notifiable Diseases Information System (*Sistema de Informação de Agravos de Notificação* - SINAN) and the Mortality Information System (*Sistema de Informações sobre Mortalidade* - SIM).

**Methods:**

Descriptive and comparative study of the SINAN and SIM information systems, was conducted via the following steps: collecting the death notices from SINAN and SIM; constructing tables and comparative graphics; and, only in scorpion sting fatalities, analyzing the distribution of deaths by age group as described in the specialized literature.

**Results:**

While SINAN identifies strong growth in the number of deaths from scorpion stings, SIM shows greater increase in the number of reported deaths from bee stings, especially in the South and Southeast regions. Notably, bees are the sole etiological agent that received more reports in SIM than in SINAN for every year in the period studied. The age-group distribution of the data on deaths from scorpion stings reinforced the indication of problems occurring in their registration in SINAN, especially since 2007, which may have an effect on analyses based on these data.

**Conclusion:**

Comparative analysis of these databases permits identification of important differences between profiles presented by these systems, which have equal relevance for Brazil as a whole and for its regions. These differences may influence the construction of various scenarios.

## Background

Brazil is one of the countries with the broadest experience in the diagnosis and treatment of envenomings by animals. These incidents, while of great interest in various fields of knowledge production and public health policy, are considered part of the group of neglected tropical diseases^1^ ^,^ ^2^ ^,^ ^3^.

In addition to the Notifiable Diseases Information System (*Sistema de Informação de Agravos de Notificação* - SINAN)^4^, which records, amongst other events, the incidence of treatment by the health services for envenoming, Brazil possesses two other national systems that offer information of related interest: deaths and hospital admissions. The Mortality Information System (*Sistema de Informações sobre Mortalidade* - SIM)^5^ aims to collect data on the total number of deaths from various causes occurring in the country, while the Hospital Information System of the Unified Healthcare Services (*Sistema de Internações Hospitalares do SUS* -SIH-SUS)^6^ records admissions to public and private hospitals. Another system that records incidents of envenoming by animals in a narrower sense is the National Poisoning Information System (*Sistema de Informações Tóxico-Farmacológicas* - SINITOX)^7^, which deals only with cases in which the Centers for Toxicological Information and Assistance (*Centros de Informação e Assistência Toxicológica* - CIATs)^7^ ^,^ ^8^provide orientation or treatment.

Over time, SINAN has improved its functioning and currently is the most used tool for analysis of envenomation cases ^4^ ^,^ ^8^. However, with respect to analyses of mortality, the SIM stands out as the most adequate system, with coverage of 90% of the deaths occurring in Brazil^9^ ^.^

Deaths caused by venomous animals must be registered concomitantly in at least both the SINAN^4^ and SIM^5^ systems. This requirement places the death notice variable in the role of a sentinel health event^10^ capable of generating a comparison between the functioning of these systems.

In order to generate quality information, a comparison between data derived from different systems and based on a specific item can reveal shortcomings and problems that might go unnoticed if only one system is examined.

Given this context, the present study aims to identify and analyze any discrepancies between SINAN and SIM in the recording of deaths caused by venomous animals between 2001 and 2015.

## Methods

This is a descriptive study comparing the SINAN and SIM information systems, following the steps below:

### 1^st^ Step: Collecting death notices from SINAN.

The data from SINAN were collected from two sources: the electronic sites of the Health Surveillance Secretariat (*Secretaria de Vigilância em Saúde* - SVS) of the Ministry of Health^11^, and the Information Technology Department of the Public Health System (*Departamento de Informática do SUS* - DATASUS)^12^ ^,^ ^13^.

The DATASUS site provides information about deaths reported to SINAN caused by the following venomous animals: snakes, spiders, scorpions and bees, organized by state, for the years 2001 to 2015. The variables related to each incident, such as “snake”, “spider”, “scorpion” or “bee”, and the resulting evolution to “death” (for the years 2001 to 2006) ^12^ or “death from reportable disease” (for the years 2007 to 2015)^13^, served as filters for data searches.

### 2^nd^ Step: Gathering death notices from SIM 

Data were also collected from the Information Technology Department of the Public Health System (*Departamento de Informática do SUS* - DATASUS)^14^ on deaths caused by venomous animals reported to SIM for the years 2001 to 2015. For this purpose, attention was focused on deaths from external causes, grouped under Category CID-10^15^, with the following codes: X20: contact with venomous snakes and lizards; X21: contact with venomous spiders; X22: contact with scorpions and X23: contact with hornets, wasps and bees.

### 3^rd^ Step: Construction of tables and comparative graphs 

Based on the data gathered in the previous steps, this stage involved the elaboration of tables and graphs comparing the SINAN and SIM systems. These examined each animal (snake, spider, scorpion or bee) separately. The graphs for the country were constructed with the same scale for all the animals in order to permit comparative analysis of the magnitude of values, which was not done in relation to regions, due to the great variability in the data. The distribution of deaths by age group was analyzed only in the case of death by scorpion sting, given that this type of incident, according to the specialized literature, is concentrated in specific age ranges, which served as a criterion for comparison of the two systems.

## Results and Discussion

Table 1 presents deaths caused by venomous animals in Brazil from 2001 to 2015, distributed by animal and information system. It is notable that the SINAN data are available from two distinct official sources, DATASUS^12^ ^,^ ^13^ and SVS^11^, without there being agreement among the presented values. It should be emphasized that checking these data at the sources also shows divergences, as the SVS data are more current. The data provided by SVS, however, are limited to three tables per animal for the entire country, its main regions and states: cases, incidences and deaths. Although it is possible to encounter studies that utilize data from SVS^16^, it is more common to find ones based on DATASUS for the elaboration of analyses of the epidemiological profile, whether for the whole country^17^ ^,^ ^18^ or for specific states or regions^16^.

**Table 1.** Deaths resulting from venomous animals, distributed by type and notification system, SINAN and SIM, Brazil from 2001 to 2005

Years	Snake			Spider		
	SINAN		SIM	SINAN		SIM
	DATASUS	SVS		DATASUS	SVS	
2001	70	69	105	9	9	5
2002	111	106	114	2	2	-
2003	123	113	122	5	4	5
2004	114	108	107	5	5	8
2005	113	100	106	9	8	13
2006	76	68	91	9	9	4
2007	117	118	90	15	15	10
2008	112	112	90	12	12	6
2009	121	125	100	15	15	6
2010	128	140	98	9	10	4
2011	131	142	96	14	15	5
2012	126	114	86	10	10	4
2013	119	120	98	14	14	6
2014	118	101	96	13	7	7
2015	92	106	104	18	12	7
Total	1671	1642	1503	159	147	90
Years	**Scorpion**			**Bee**		
	SINAN		SIM	SINAN		SIM
	DATASUS	SVS		DATASUS	SVS	
2001	43	39	33	5	5	41
2002	57	48	40	14	13	49
2003	51	49	39	7	7	38
2004	42	40	37	9	9	47
2005	48	45	40	13	12	48
2006	28	24	21	13	13	55
2007	61	61	31	18	33	53
2008	85	85	43	11	11	40
2009	90	89	32	33	32	67
2010	64	74	26	26	27	52
2011	78	82	25	29	28	70
2012	89	87	30	30	30	54
2013	73	73	24	37	37	62
2014	99	88	33	34	31	74
2015	77	118	26	33	36	70
Total	985	1002	480	312	324	820

When comparing SINAN data with those from SIM, it can be observed that, except for bees, SINAN presents a higher number of deaths resulting from venomous animals (Table 1). This result was unexpected, given that SIM aims to record data on the totality of deaths occurring in Brazil^5^, while SINAN restricts itself to notifications of cases in which the health services attend the victim^4^. This means that SIM can accept records of deaths that were not attended by the health services, which logically would make the SIM total greater than that of SINAN. How, then, to explain the number of cases in which health services were involved, but were not recorded by the system that records mortality? In his analysis of deaths resulting from scorpions in Rio de Janeiro and recorded by SIM and SINAN for the period 2001 to 2015, Souza (2018) ^19^ confirmed the existence of case duplication and data entry errors on the part of SINAN, which overstated the number of deaths. On the other hand, he observed incorrect codification of the basic causes of death by SIM, which led to underestimation of the number of deaths. At the beginning of his study, he identified 18 deaths based on notifications in SINAN and 10 in SIM. Following analysis and investigation, there was an inversion and the final confirmation numbers were 11 (SINAN) and 13 (SIM), respectively.

It is quite possible that the same problems identified for the state of Rio de Janeiro occurred in Brazil as a whole, which might explain the differences encountered in the data from SIM and SINAN. Currently, however, there are no studies of this type for any other state, nor even any adequate study for the whole country.

Comparison of the data on deaths caused by the four etiologic agents reveals additional important differences.

**Figure 1.**
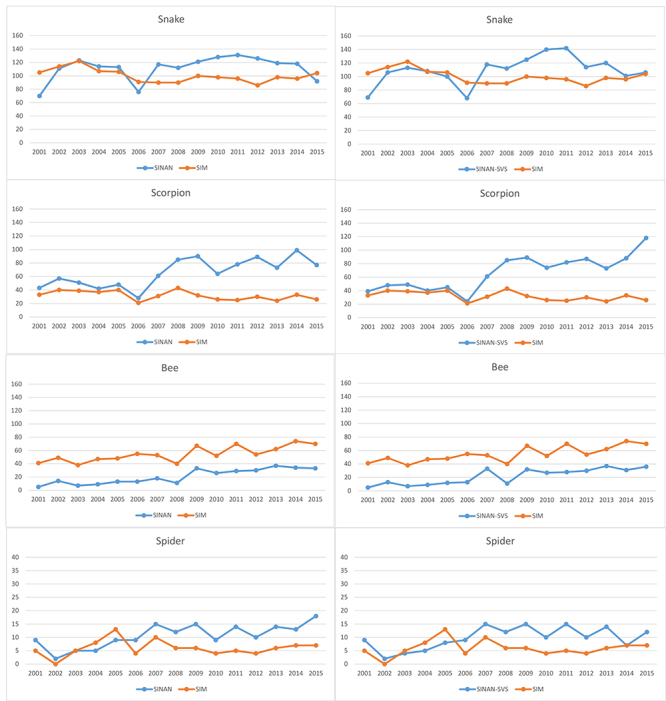
Deaths caused by venomous animals in Brazil from 2001 to 2015, distributed by information system, for each species.

The graphs in Figure 1 referring to snakes, scorpions and spiders reveal that the greatest differences between the SINAN and SIM systems occurred starting from 2007. It needs to be emphasized that this was the year when the SINAN notification form^20^ ^,^ ^21^ underwent changes that required dividing its data into two periods: 2001 to 2006^12^ and 2007 onward^13^.

It is necessary to remember that the variable “evolution” for the period 2001 to 2006 encompassed the following categories: 1 - cured, 2 - cured with after effects, 3 - death, and 9 - unknown. From 2007 forward the variable “evolution” came to contain these categories: 1 - cured, 2 - death from notifiable disease, 3 - death from other causes and 9 - unknown^20^. It should be noted that filling in this field of the form is deemed essential, but not obligatory^21^.In the notification form for 2007, which became compulsory in 2010, the physical closeness of field 56, which deals with work-related accidents, and field 57, dealing with the evolution of the case^20^ ^,^ may constitute one among other sources of errors. In both fields there are options identified with the number 2. Field 56 offers 1 - yes, 2 - no, and 9 - unknown^20^. This proximity could foster the repetition of the number 2 as indicating a negative evolution of the case, ending in death. This situation could be a factor influencing the larger number of deaths recorded by SINAN, beginning in 2007, to the detriment of SIM. This was identified in the seminar for personnel of state programs for surveilling cases of animal poisoning that took place in Niteroi in 2017^22^, and verified by Souza (2018)^19^ in his investigations on deaths due to scorpionism in the State of Rio de Janeiro. The author identifies a sequence of errors and fragilities along the flow of data collection and feeding of health information systems. These errors ranged from the poor completion and incompleteness of the forms by basic services personnel and typing errors such as duplication of cases as well as the episodes in relation to fields 56 and 57 of the SINAN compulsory notification form.

The evolution of envenomings that result in deaths show marked differences related to the etiological agents involved. Deaths due to scorpions stings are predominantly early, whereas in severe cases attributed to snakes and bees late complications that will influence the fatal outcome are observed ^3^ ^,^ ^8^ ^,^ ^16^ ^,^ ^17^ ^,^ ^18^ ^,^ ^19^ ^,^ ^21^ ^,^ ^25^. These differences may result in heterogeneous time intervals in the recording of final case results that can promote faster notification to SINAN in lethal scorpionism, and help to explain some of the apparent prioritization of SIM in cases of late deaths after massive contact with bees.

Deaths induced by venomous animals are not included in the official list of preventable causes^19^, which, associated with local difficulties in investigating and analyzing each individual case, may also influence the notification discrepancies identified in the present study.

Apart from the numerical differences observed, each system presents differing tendencies between snake and scorpion poisonings. While SINAN indicates a clear trend toward growth in scorpion cases and a lower profile for snakebites, SIM paints a picture of apparent stability for both (Figure 1). This finding raises the alarm in relation to the production of skewed analyses when a single system is used, and the need to take this into account when evaluating results previously published.^17^ ^,^ ^18^

**Figure 2.**
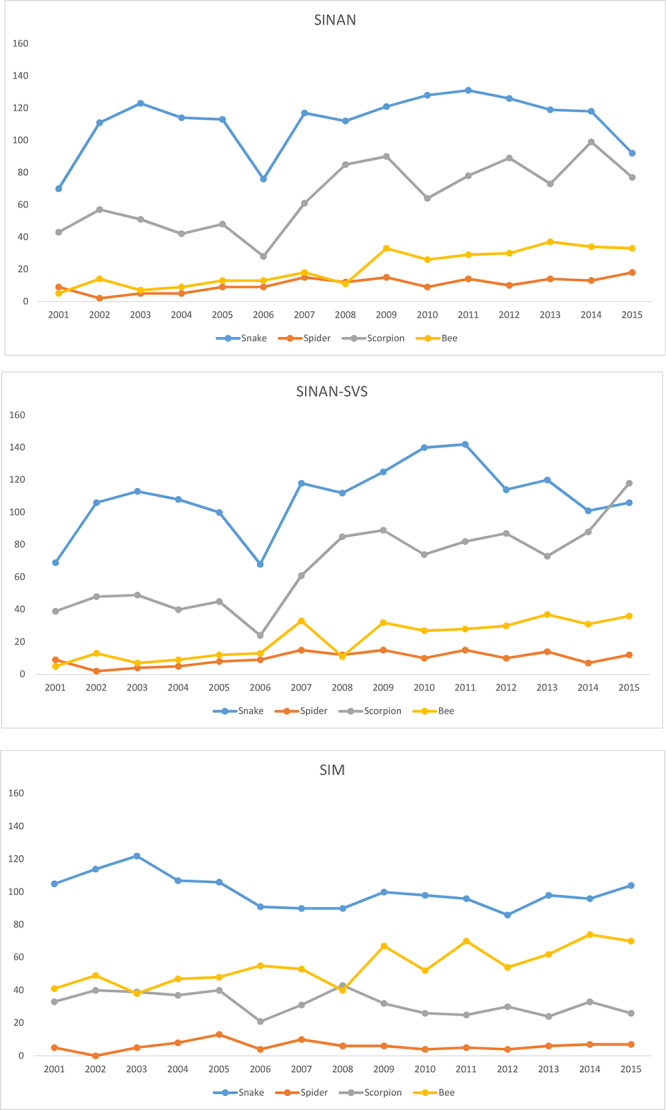
Deaths caused by venomous animals in Brazil from 2001 to 2015, distributed by species for each information system

Figure 2 presents the mortality patterns of the four animals in each system. Comparison accentuates the differences observed between SINAN and SIM. For the latter, deaths from bee stings show the general tendency to increase, moving into second place in ranking. It should be noted that this is the sole etiologic agent that does not possess a specific serum for its treatment. Analysis of the various SINAN sources highlights the recent sharp increase in deaths from scorpion stings, which, according to the SVS, have become the leading cause in this respect, a fact not recorded by DATASUS.

Once again, there is confirmation that the use of a single system may generate a weak epidemiological profile that is incapable of closely reflecting the reality of the situation under observation.

**Figure 3.**
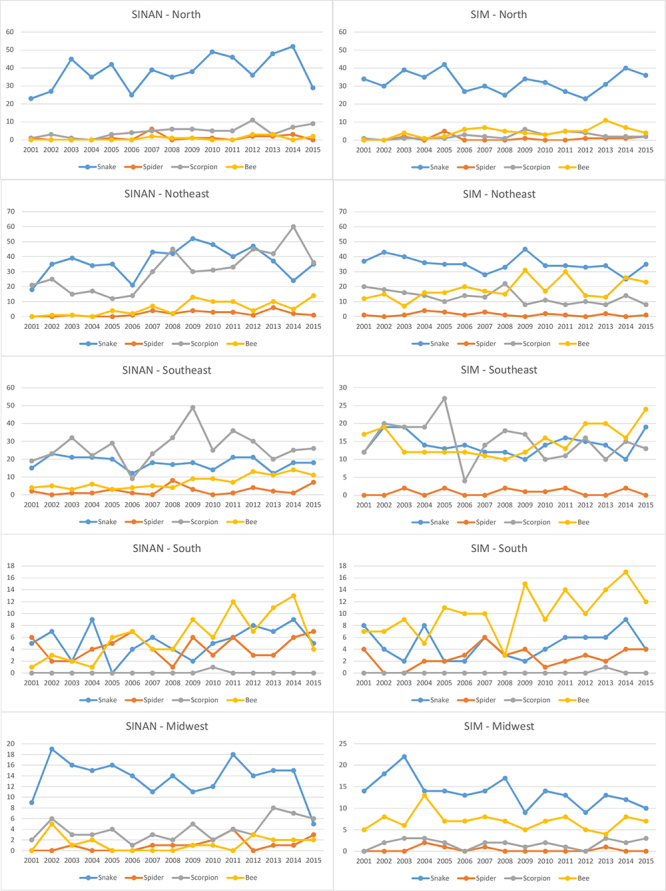
Deaths caused by venomous animals, distributed by species for each information system, by region, from 2001 to 2015.

Analysis by region also reveals marked differences between the two systems. Although the North, Northeast and Center-West present profiles similar to that of the country as a whole, reproducing the same observed discrepancies between the two systems, the Southeast region stands out with the highest number of deaths from scorpions, as is also the case in the SIM. Except for 2006, this pattern was observed in SINAN for the whole period, and in SIM from 2001 and 2009. Notably, the Southeast region presents an inversion in the epidemiological profile: in 2009 the number of deaths from bee stings surpassed those from snakes and, in 2010, those from scorpions, making bees responsible for the majority of deaths recorded by SIM, with the exception of the year 2011. An even more divergent profile was observed for the South region, in which the deaths due to bee stings were more frequent for SINAN, beginning in 2005, with the exceptions of 2007, 2008, 2012 and 2015, as well as for SIM during the whole period, with the exceptions of 2001 and 2004 (Figure 3). The significant number of deaths due to massive attacks by Africanized honeybees fully justify Brazilian efforts to develop a new apilic antivenom ^23^. After the clinical trials recommended by ANVISA, this new immunobiological should be available through the Brazilian SUS network ^22^ ^,^ ^24^.

**Figure 4.**
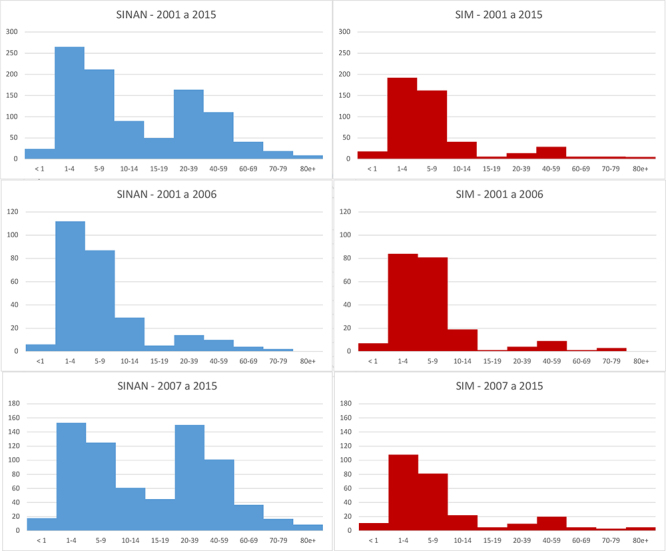
Deaths due to scorpion stings in Brazil, distributed by victim age group for the periods 2001 to 2006, 2007 to 2015 and 2001 to 2015, according to the SINAN and SIM information systems.

Figure 4 compares SINAN and SIM data on deaths from scorpion stings, distributed by age group, through three time periods: 2001 to 2015, 2001 to 2016 and 2007 to 2015. The SINAN data used in this figure come from DATASUS, as the SVS does not provide data by age. Deserving of attention, for the period from 2001 to 2015, is the difference in age distribution between the two systems, especially the concentration of deaths reported in SINAN for age groups above 20 years.

Continuing the analysis of these data reveals that this pattern is not present for the period 2001 to 2006, for which the two systems present a data distribution similar to that reported in the literature on scorpion deaths, with the main concentration of deaths in age groups below 14 years^25^. In the period 2007 to 2015, however, this distribution changed considerably with the appearance of a high frequency of deaths in age groups that are not commonly victims of such events, even to the extent of showing the same number of deaths of young adults and infants. SIM does not identify this change, which reinforces the previous observation referenced in Figure 1 that the alterations shown by SINAN may have been influenced by the 2007 introduction of the new notification form.

## Concluding remarks

Comparative analysis of deaths caused by venomous animals registered by SINAN and SIM in the period 2001 to 2015 permits the identification of important differences between the profiles outlined by these systems, which may influence the construction of various scenarios. These findings support our suggestion that future studies on deaths due to venomous animals in Brazil must compare the different information systems in order to contextualize investigations within the discrepancies found between their databases.

In contrast to the strong tendency for SINAN to demonstrate growth in the number of scorpion-induced deaths, SIM indicates that the number of deaths from stings is bee growing the fastest. It is noteworthy that bees are the only etiologic agent to receive more notifications in SIM than in SINAN for all years of the period studied but do not possess a specific serum for treatment.

The age-group distribution of deaths caused by scorpions highlights that the problems experienced in SINAN as to accurate reporting may affect analyses based on this system’s data.

The strategic importance of information systems for decision-making in health service routines, public policies and scientific research highlights the need for periodic analysis and evaluation, in order to achieve constant improvement. This commitment reinforces the importance of the permanent training, supervision and continuous education of teams involved at all levels, ranging from those responsible for collecting the primary data to high-level managers.

### Abbreviations

Not applicable.
